# PSYCHOSOCIAL DISADVANTAGE DURING CHILDHOOD AND MIDLIFE HEALTH

**DOI:** 10.1093/geroni/igae098.0556

**Published:** 2024-12-31

**Authors:** Ryan Brown

**Affiliations:** Texas Tech University, Texas, United States

## Abstract

Psychosocial disadvantage in childhood contributes to health disparities in aging-related health outcomes through biological and behavioral pathways. We leveraged the 30-year longitudinal NHLBI Growth and Health Study to examine associations between (a) low childhood SES (age 10) and (b) perceived stress in childhood (age 11) with midlife insulin resistance and epigenetic aging (age 40) in a sample of 433 Black and white women. We also explored whether late adolescent (age 19) waist circumference would mediate the relationships between SES and perceived stress and aging-related outcomes. Epigenetic aging was assessed in saliva and quantified through the GrimAge2 and DunedinPACE clocks. Insulin resistance was represented by the Homeostatic Model Assessment of Insulin Resistance (HOMA-IR). For each aim, multivariable linear regression models were estimated. Covariates included age, race, diabetes status, smoking status for all outcomes, and analysis cluster and estimated cell type composition variables for epigenetic clocks only. Higher parental education was associated with lower insulin resistance (b=-0.22, SE=0.10, p=.03), lower midlife GrimAge (b=-1.76 years, SE=0.56, p=.002), and slower midlife DunedinPACE (b=-0.03, SE=0.2, p=.04). Childhood perceived stress was significantly indirectly associated with midlife insulin resistance, b=0.01, 95% CI [0.001,0.01], p=.02, and midlife GrimAge, b=0.016 years, 95% CI [0.003,0.04], p=.01, through late adolescent adiposity. This highlights preliminary evidence for this life-course perspective on psychosocial stress, adiposity, and aging-related health. Future work may examine these individual factors in the context of broader structural systems (e.g., schools) and other facets of SES to better understand area-level effects (e.g., familial, neighborhood, region) for tailoring social interventions.

